# The Conforming Brain and Deontological Resolve

**DOI:** 10.1371/journal.pone.0106061

**Published:** 2014-08-29

**Authors:** Melanie Pincus, Lisa LaViers, Michael J. Prietula, Gregory Berns

**Affiliations:** Emory University, Atlanta, Georgia, United States of America; University of Bologna, Italy

## Abstract

Our personal values are subject to forces of social influence. Deontological resolve captures how strongly one relies on absolute rules of right and wrong in the representation of one's personal values and may predict willingness to modify one's values in the presence of social influence. Using fMRI, we found that a neurobiological metric for deontological resolve based on relative activity in the ventrolateral prefrontal cortex (VLPFC) during the passive processing of sacred values predicted individual differences in conformity. Individuals with stronger deontological resolve, as measured by greater VLPFC activity, displayed lower levels of conformity. We also tested whether responsiveness to social reward, as measured by ventral striatal activity during social feedback, predicted variability in conformist behavior across individuals but found no significant relationship. From these results we conclude that unwillingness to conform to others' values is associated with a strong neurobiological representation of social rules.

## Introduction

A person's set of values helps define one's individual and group identity. Of particular importance in defining one's identity are protected, or sacred, values—those defended resolutely and not compromised for material tradeoffs [1]. These include core religious beliefs and moral norms that constrain decision-making across a person's lifetime. In many cultures, violating sacred values is tantamount to disavowing group membership [2], underscoring the importance of sacred values to group identity.

Research demonstrates that individuals process sacred values deontologically, whereby individuals construct and represent these values according to a rule-based schema of rights and wrongs, irrespective of context or outcome [1], [3], [4]. Evidence suggests that individuals differ in the degree to which they endorse deontological rules [5], [6], and individuals with a deontological disposition may have a stronger neurobiological representation of deontological rules. Consequently, we hypothesize that they may be more unwilling to bend the rules of right/wrong in the face of social influence. This hypothesis is supported by the finding that the certainty with which an attitude is held is correlated with greater resistance to social influence [7]–[9]. Accordingly, individuals with a strong deontological disposition who readily appeal to absolute rules that confer greater certainty should be expected to be less conformist generally. Because sacred values are categorical in nature, it would be difficult to test this hypothesis and discern individual differences in deontological disposition using behavioral measures alone. Alternatively, neuroimaging offers a way to assess variability in deontological resolve across individuals.

Our previous neuroimaging study supported the hypothesis that sacred values are processed as deontological rules in the brain, evidenced by activation in the ventrolateral prefrontal cortex (VLPFC) during the passive processing of sacred value statements [10]. The VLPFC has been implicated in rule-based decision-making [11]–[14], and imaging studies designed to isolate the process of rule retrieval consistently demonstrate VLPFC activation [15]–[18]. We contend that the VLPFC is recruited in the natural representation of sacred attitudes and values because these constructs tap deontological rules about the way things ought to be.

Importantly, our previous study found that activity in the VLPFC was localized to the left hemisphere, a laterality finding that is consistent with evidence suggestive of the left hemisphere's role in maintaining stable mental representations and counteracting belief revision [19]–[21]. In a recent study, participants resisted updating their beliefs in the face of unfavorable information in a control condition, whereas application of transcranial magnetic stimulation to the left VLPFC—but not the right VLPFC—facilitated belief updating of unfavorable information among participants in the experimental condition [22]. Taken together, these findings suggest that the left VLPFC may be implicated in the representation of sacred values, relative to non-sacred values, because this region accesses deontological rules that resist revision. Our previous study also found that activity in the left VLPFC for sacred values was significantly correlated with the participants' level of involvement in social groups, suggesting that the neurobiological representation of deontological rules may be modulated by social considerations. Across individuals, then, relative activation in the VLPFC may indicate how strongly an individual represents deontological rules and predict (un)responsiveness to social feedback. To the degree that individual differences in conformity are driven by how strongly one represents deontological rules, we predict that greater activation in the VLPFC during the passive retrieval of sacred values should be associated with less conformist behavior in our current study.

Because prior research suggests that the reward system is implicated in responding to social feedback (see [23] for a review), we were also interested in examining whether social feedback concerning one's value system is processed by the brain's reward system and motivates conformity. Prior studies suggest that the ventral striatum (VS) may provide a positive/negative reinforcement signal indicating degree of consensus with group opinion [24], [25], and that this signal may predict individual variability in conformist behavior [24]. However, these studies gauged the extent to which participants conformed their ratings of the hedonic qualities of stimuli such as faces and t-shirts, the results of which may not necessarily apply to the pliability of one's personal value system. To examine the possibility that (dis)agreement with others' values provides a meaningful reinforcement signal, we tested whether activity in the VS during the presentation of social feedback was modulated by the magnitude of social consensus and/or varied systematically with individual differences in conformist behavior. If the magnitude of agreement between one's set of values and others' provides a meaningful reinforcement signal, activity in the VS should correlate with the magnitude of social consensus and predict conformist behavior across individuals.

To answer these questions, we ran two experiments that were similar in design to our previous study of sacred values [10], but with an added social feedback treatment that provided information on the popularity of a participant's set of values. We gauged how strongly the feedback influenced the participants' subsequent behavior in an auction to measure individual differences in conformity. The present findings confirm our hypothesis that a stronger neurobiological representation of sacred values is correlated with weaker conformist tendencies.

## Methods

### Participants

Seventy-seven adult participants were recruited from Emory University campus to participate in the study; forty-one participated in the first experiment (M = 23.6 years, SD = 7.6), and thirty-six participated in the second experiment (M = 23.5 years, SD = 5.5). Four participants were removed from the data analysis, three due to excessive motion and one due to an error recording responses. All participants provided written informed consent and reported no history of psychiatric or neurological disorders. Participants received $40 base pay for participating in the study with the chance to earn additional money in the auction phase. The study was approved by the Emory University Institutional Review Board.

### Procedure

The study consisted of four phases, with brain imaging data collected during the first three phases. Stimuli were predetermined value statements embodying values whose likely importance to the participants varied to greater and lesser extents. Statements ranged from simple preferences (e.g. “You are a white wine drinker.”), asserted beliefs (e.g. “You believe in God.”) and stances on actions (e.g. “North Korea should be nuked.”). Each value statement had its opposite included in the stimulus set (e.g. “You are a red wine drinker.”), for a total of 62 pairs of opposing value statements in the first experiment. The second experiment retained 60 of the 62 pairs and included four new pairs, for a total of 64 pairs. The stimuli were presented in random order in each phase. In the first phase, the passive phase, value statements were passively presented one at a time for three seconds, after which the participant pressed a button in a self-paced manner to advance to the next statement. In the second phase, the active phase, participants were presented with the opposing value statement pairs and instructed to choose the statement from each pair that they most agreed with.

**Figure 1:**
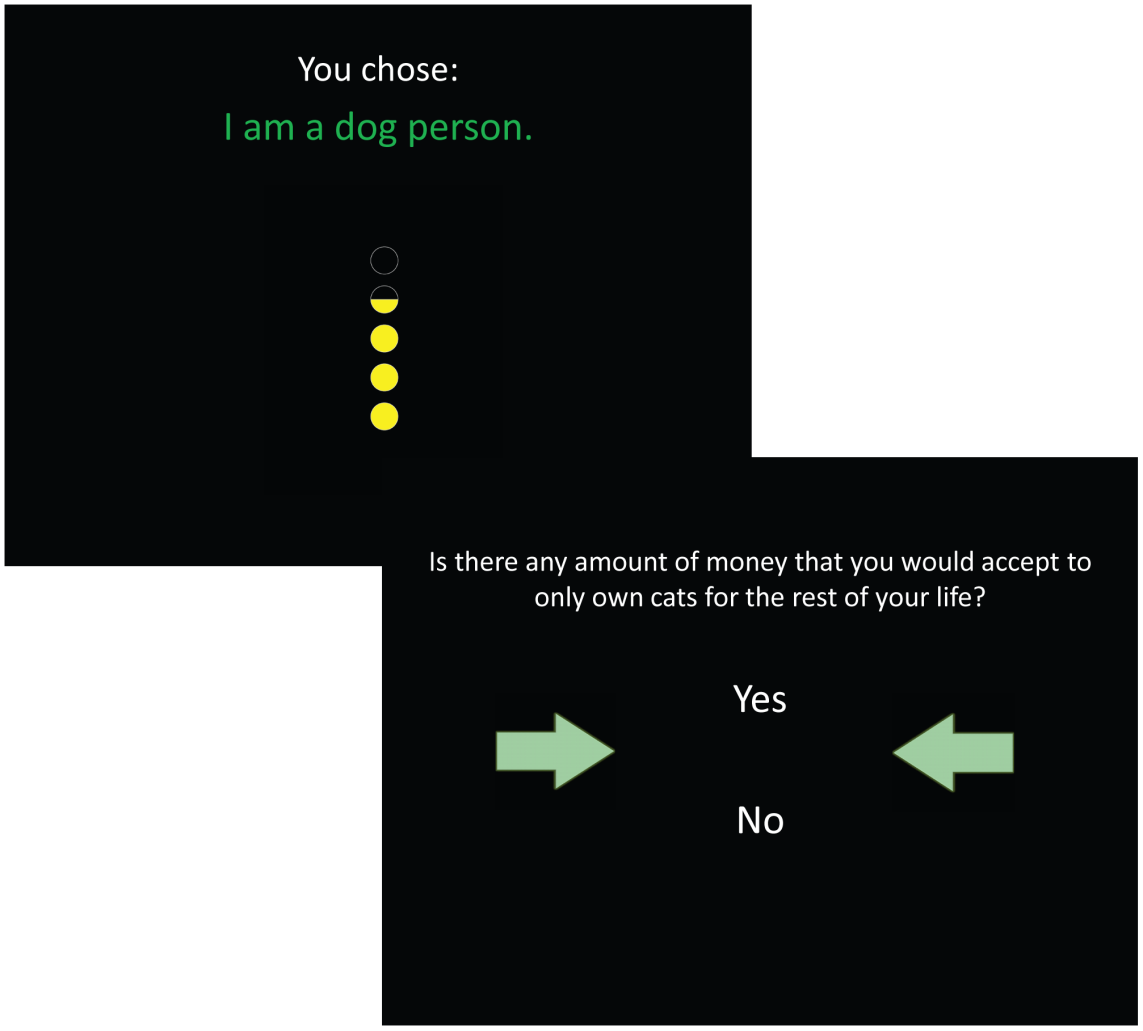
Trial Structure for the Hypothetical Phase. On each trial, a participant viewed the value statement they chose in the active phase along with social feedback indicating the percentage of past participants who chose the same value statement. For the participants in the second experiment, the feedback was comprised of five circles and each filled-in half circle signified 10% past participant agreement. This feedback was presented for a fixed period of two seconds, after which a participant pressed a button to advance to the next screen when ready. On the next screen, a participant was presented with a hypothetical question asking if they would be willing to accept money in exchange for endorsing the opposing value statement. The participant answered by choosing “yes” or “no” in a self-paced manner.

In the third phase—the hypothetical phase—participants were presented with each of their chosen value statements (from the active phase) along with social feedback indicating the percentage of past participants who chose the same value statement. The percentages were calculated from choices made by participants in our previous study [10]. In the first experiment, the feedback was comprised of five circles with each half filled-in circle representing ten percent (see Figure 1). In the second experiment, the participants viewed one of five possible sentences that approximated the magnitude of the social consensus (e.g. “About half of the participants agreed with you.” corresponded to a range of 40–60% consensus). After being provided social feedback, participants were posed a hypothetical scenario asking if they would be willing to accept money to disavow their previously chosen values and accept its opposite. For example, if a participant chose “I am a dog person,” the hypothetical question posed would be “Is there any amount of money you would accept to only own cats for the rest of your life?”

In the fourth phase, which took place outside of the scanner, the Becker-DeGroot-Marshak (BDM) auction mechanism was used to assess the sacredness of the participants' chosen values [26]. For each value, the participants were asked to submit an asking price between $1 and $100 that they would be willing to accept in exchange for providing their personal signature endorsing the opposing value at the end of the auction. When participants chose to opt out of the auction for a given value, they signaled deontological resolve towards the value by refusing to compromise it for money. Optout value statements were considered sacred, whereas values that participants bid on were considered non-sacred.

After submitting bid decisions for the entire set, the participants rolled a pair of 10-sided dice, the purpose of which was to randomly determine the price offered for each non-sacred item in the auction. One die represented a multiplier of $10, the other die a multiplier of $1, such that each dice roll could randomly generate a price between $1 and $100. If the dice roll was higher than the asking price, the participants received an amount of money equal to the dice roll, and the opposing value was added to the final set of value statements to be endorsed. If the dice roll was lower than the asking price, participants received no earnings. To calculate participants' total earnings, the amount of money obtained across all trials was averaged—with no money earned on optout trials and trials in which the dice roll was lower than the asking price. The final set of value statements was printed on a document that participants were required to sign.

A separate Tobit regression was run to calculate each participant's social conformity score [27]. Four participants were excluded for lack of sufficient variability in their bid submissions. The 60 value statement pairs that appeared in both experiments were the only ones included in the regression model. The equation for the Tobit model included the following variables:
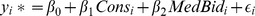
where y_i_* is a latent variable representing how much money the participant would require to endorse an opposing value in the BDM auction. Opting out of the auction indicates that the value statement is worth more to the participant than the alternative of compromising it for $100. Because the true worth of these sacred statements cannot be accurately gauged (i.e. given the $100 auction bid limit), they were assigned a monetary worth of $101 and the model was informed to censor any trials with bid prices above $100.

*MedBid* corresponds to a statement's median bid price. For each value statement, the median bid price was calculated from the range of auction bid prices submitted across all participants in our previous study of sacred values [10]. Each statement's median bid price was included in the model because participants are likely to submit bids that reflect the inherent worth of the statement. For example, the median bid prices for the value statements “I am a MAC person.” and “I am a PC person.” were $10 and $3 respectively because this statement pair is inherently mundane and lacking in sacredness. Because this earlier study did not include social feedback, the contribution of each value's inherent worth to the bid price could be isolated and added to the Tobit model.

The variable *Cons* represents the percentage of previous participants who chose the same value as the participant. The beta value for *Cons* indicates how strongly the social feedback modulated the worth a participant assigned to value statements in the BDM auction and was used to represent each individual's conformity score. To generate comparable conformity scores across the experiments, the magnitude of social consensus was entered as a continuous variable taking on a range of numerical values between zero and ten. In the first experiment, each half-circle represented ten percent social consensus and was scaled to fit the range zero to ten; for example, 70% social consensus was presented as seven half-circles in the hypothetical phase and was entered as a seven in the regression. Social consensus in the second experiment was mapped onto the same scale by choosing the median value of social consensus represented by each statement and scaling it to the range of zero to ten. For example, the statement “About half of the participants agreed with you” was represented by the numerical value of five because the statement corresponded to a range of 40–60% social consensus, with 50% as the median numerical value.

### Imaging Acquisition and Analysis

Neuroimaging data were acquired using a 3T Siemens Magnetron Trio whole body scanner (Siemens Medical Systems, Erlangen, Germany). Four functional runs were recorded, the first experiment consisting of 62 trials per run and the second consisting of 64 trials per run. The first two functional runs corresponded to the passive phase, the third run to the active phase, and the fourth run to the hypothetical phase. The trials lasted a variable duration depending on participants' decision time. The functional data involved the acquisition of 33 axial slices of 3.5 mm thickness with a matrix size of 64×64 over a field view of 192 mm (T2*-weighted, TR = 2000 ms, TE = 30 ms). T1-weighted structural images were also collected (TR = 2300 ms, TE = 3.4 ms, flip angle = 8°, 240×256 matrix, 176 sagittal slices, 1 mm^3^ voxel size). The data were analyzed using Analysis of Functional NeuroImaging (AFNI) program (http://afni.nimh.nih.gov/afni/). The functional data were slice-time and motion-corrected, spatially smoothed with a 8-mm Gaussian smoothing kernel, aligned to the anatomical images, and affine transformed into Talairach space. General linear models (GLMs) of the passive and hypothetical neuroimaging data were created for the fifty-eight participants who had valid conformity scores and a set of sacred and non-sacred value pairs.

Because we could not rule out the possibility that social feedback in the hypothetical phase may have influenced whether a participant chose to bid or opt out in the BDM auction, we sought to identify a set of values that would be invariably classified as sacred and non-sacred across participants, social feedback notwithstanding. We singled out a set of values that was consistently categorized as sacred and non-sacred by the majority of participants across our current and prior study [10]. The subset of sacred values included 11 pairs that were opted out in the auction by more than 60% of participants in our prior study and at least 60% of participants in one of the two experiments in the current study. The subset of non-sacred values included 11 pairs bid on in the auction by more than 60% of participants in the prior and current study. Table S1 includes a table of the set of sacred and non-sacred items used in the GLM of the neuroimaging data for the passive phase. If a participant opted out on a value statement that is a member of the sacred subset, this statement and its opposite were included in the GLM model of the participant's passive phase as sacred/chosen and sacred/not chosen, respectively. For example, if a participant chose “You do not like to hurt animals.” and opted out on the statement in the auction, it was categorized as sacred/chosen in the participant's model because it was a member of the sacred subset, and the opposing statement—“You like to hurt animals.”—was categorized as sacred/not chosen. If a participant bid on a statement in the BDM auction that was a member of the non-sacred subset, this statement and its opposite were included in the participant's model as non-sacred/chosen and non-sacred/not chosen, respectively. For each participant, then, twelve regressors were included in the passive phase GLM. These included 1) sacred/chosen values, 2) sacred/not chosen values, 3) non-sacred/chosen values, 4) non-sacred/not chosen values, and six motion parameters along with a constant and linear drift term for each run. All task-related regressors were modeled as variable duration events and convolved with a canonical hemodynamic response function.

The analysis of the passive phase was focused on a region of interest (ROI) in the left VLPFC defined by our earlier study of sacred values [10]. This ROI of the left VLPFC (177 voxels) was defined by the contrast of optout > bid statement pairs among participants in our original study, thresholded at *p*<0.001 with a cluster threshold of k≥53. The cluster threshold was determined using the AlphaSim routine in AFNI and yielded a whole-brain familywise error rate (FWER) of 0.05. For each participant in the current study, the beta coefficients for the regressors were extracted and averaged across all voxels in the left VLPFC ROI. Using SPSS, the beta coefficients for the contrast of chosen sacred > chosen non-sacred were entered into a GLM as the dependent variable. The participants' conformity scores were entered as the independent variable of primary interest, but we also included a dummy variable for the experiment and an interaction term between the dummy variable and the conformity score. These latter two terms were included to control for possible effects associated with the difference in social feedback format between the two experiments. Because these terms were not found to be significant in the GLM, as explained in the results section below, and the experimental design was otherwise identical between the two experiments, the participant data from the two experiments were analyzed together. To confirm that the results from our earlier study replicated, we also ran a GLM to test whether the beta coefficients from the less constrained contrast of sacred (chosen and not chosen) > non-sacred values (chosen and not chosen) were significantly different from zero, and controlled for possible differences between the two experiments by entering the dummy variable as a fixed factor.

In the hypothetical phase, the trials presented chosen value statements from the active phase along with social feedback indicating the percentage of past participants who agreed with the participants' value statement choices. In the neuroimaging GLM for this phase, we included one task-related regressor that modeled the onset of the social feedback for each trial. We also included a parametric modulator to model the magnitude of the social consensus. The numerical values representing the magnitude of social consensus were the same as those used in the Tobit regression described above, i.e. the modulator took on a value from zero to ten. The six motion regressors and a constant and linear drift term for each run were also included in the model. Using an anatomical ROI of the bilateral VS modified from the standard Talairach atlas provided by AFNI (133 voxels), we extracted the beta values for the regressor modeling the social feedback modulated by the magnitude of consensus, and calculated the mean beta value across the ROI. We performed a GLM to test whether activation in the striatum was significantly modulated by the magnitude of social consensus and whether striatal activity was significantly predicted by individual differences in social conformity. As with the model in the passive phase, we entered the beta values from the striatal ROI as the dependent variable and the conformity scores as the independent variable, controlling for differences between the experiments by entering an experimental dummy variable and an interaction term between the experimental dummy and conformity scores.

## Results

The social feedback provided across all participants in the hypothetical phase had a left-skewed distribution in the two experiments (M = 72%, SD = 25% in the first experiment; more than 60% of the feedback trials in the second experiment indicated majority consensus: “Participants very often agreed with you.” or “All of the participants agreed with you.”). The distribution of asking prices across participants in the BDM auction was bimodal, with the majority of prices falling into the $1 or optout bin. In addition to the $40 base pay, the participants earned an average of $26.82 from the BDM auction. The vast majority of participants had positive social conformity scores, indicating that as social consensus increased, participants submitted higher asking prices in the BDM auction.

**Figure 2:**
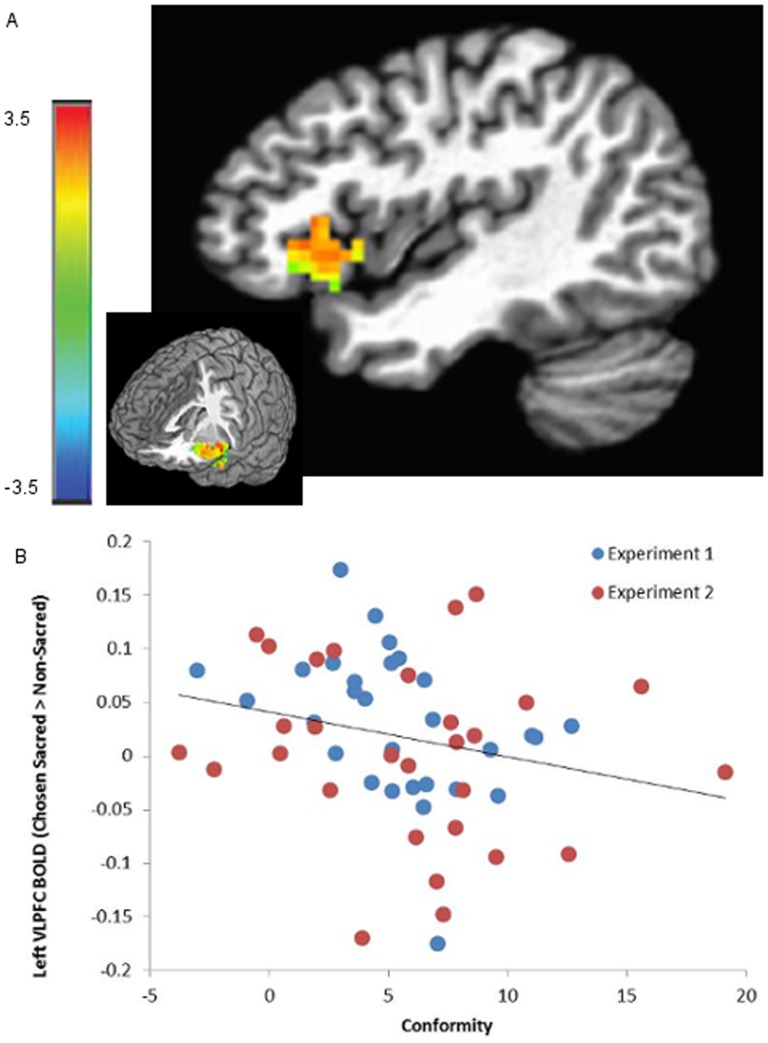
Individual Differences in Conformity and Neural Responses to Chosen Sacred > Non-sacred Values. A: The left VLPFC ROI was created from the contrast sacred > non-sacred values in our prior study [10]. The map was thresholded at p<0.001 to define the ROI. The intensity of the colors represent the t-statistic from the contrast chosen sacred > non-sacred in the current study (peak MNI coordinates: x = −40, y = 23, z = −7). B: Participants' conformity scores were negatively correlated with VLPFC activation for chosen sacred > non-sacred values, *F*(1,56) = 4.198, *p* = 0.045, β = −0.002.

We replicated the findings from our prior study of significantly greater activation in the left VLPFC for sacred > non-sacred values, *F*(1,56) = 11.384, *p* = 0.001. We also found that there was significantly greater activation in the left VLPFC ROI for the constrained set of chosen sacred vs. non-sacred values, *F*(1,56) = 7.301, *p* = 0.009. As predicted, there was a significant negative relationship between activity in the left VLPFC for chosen sacred > non-sacred values and participants' conformity scores, *F*(1,56) = 4.198, *p* = 0.045, β = −0.002 (see Figure 2). The stronger a participant's VLPFC activation in the chosen sacred > non-sacred contrast in the passive phase, the less socially influenceable the participant behaved in the BDM auction. Conversely, the weaker the activation in the VLPFC for the chosen sacred > non-sacred contrast, the more the participants' choices in the auction were influenced by social feedback. There were no significant differences in left VLPFC activation for chosen sacred > non-sacred between the two experiments, as evidenced by the insignificance of the experimental dummy variable, *F*(1,56) = 2.482, *p* = 0.121. Furthermore, the difference in social feedback format between the two experiments did not significantly influence the relationship between VLPFC activation and social conformity, as reflected by the insignificance of the interaction term between the experiment and conformity scores, *F*(1,56) = 1.157, *p* = 0.287.

ROI analysis of the ventral striatum was conducted to examine whether feedback indicating the popularity of a participant's set of values provided a meaningful reinforcement signal and/or correlated with conformist behavior. Across all trials in the hypothetical phase, the VS was not significantly modulated by the magnitude of social consensus, *F*(1,56) = 0.121, *p* = 0.730. Furthermore, the beta values from this ROI analysis did not have a significant relationship with the individual conformity scores, *F*(1,56) = 0.067, *p* = 0.797.

## Discussion

The majority of participants had positive conformity scores, indicating that they raised their bid prices and/or opted out as the consensus increased. Thus, most participants were impelled to place higher worth on their values when they received feedback that the majority of past participants agreed with them. Conversely, when the consensus position was attenuated, conformist individuals placed lower worth on their values. That the social feedback variable was positive in the majority of participants' Tobit models is a particularly striking testament to the strength of social influence, given that raising one's bid prices leads to reduced earnings overall.

We replicated the finding from our prior study that activation in the left VLPFC was significantly greater for sacred vs. non-sacred values in the passive phase. In our prior study, we evaluated alternative models and found that VLPFC activation was significantly associated with sacred values after controlling for variability in semantic richness, statement length, syntax, and legality of the value statements [10]. Given that we controlled for various confounds in our prior study, and replicated the result in our current study, we feel confident that the left VLPFC is strongly associated with the retrieval of sacred values.

The results of our current study suggest that individual differences in conformity are predicted by deontological resolve. The left VLPFC has been demonstrated to be involved in rule retrieval and resisting belief revision, making it well suited to uphold deontological rules with rigidity [15]–[22]. It follows that greater activity in the left VLPFC region across individuals may indicate a more rigid endorsement of deontological rules, whereas weaker activity may indicate less reliance on deontological rules and greater flexibility. The finding that activity in the left VLPFC for chosen sacred > non-sacred values negatively correlates with individual differences in conformity supports the hypothesis that stronger deontological resolve predicts less conformist behavior with respect to values and attitudes relevant to one's self-concept. Conversely, weaker activity in the VLPFC predicts relatively stronger conformist behavior, perhaps owing to weaker representation of deontological rules and greater flexibility in one's value system when confronted with social influence.

To the extent that normative concerns impact individual differences in conformity, we expected to find that striatal activation fluctuated with the magnitude of social consensus and that this activity had a significant relationship with individual differences in conformity. Across all trials in the hypothetical phase, we found that social feedback did not significantly modulate activity in the VS, nor did striatal activity correlate with individual differences in conformity. Our results stand in contrast with other studies that have demonstrated a relationship between striatal activation and responsiveness to social influence [24]–[25]. These studies measured how participants modified their judgments of stimuli like songs and t-shirts, in which case striatal activity may be associated with processes involved in re-appraising the hedonic value assigned to the stimuli, so as to cohere with group consensus. Alternatively, striatal activity in these contexts may reflect mechanisms associated with normative social influence—as opposed to informational social influence—whereby agreement with others provides socially rewarding feedback. We used a class of stimuli that were lacking in hedonic qualities but were likely relevant to each participant's sense of identity. Striatal activity may not meaningfully respond to social consensus because there is not a re-appraisal of the hedonic value of the stimuli, or normative social influence may not bear heavily when it comes to personal attitudes. Informational social influence may dominate how conformist individuals behave in the domain of personal attitudes, and processes associated with this type of influence may not depend on the striatum.

A limitation of this study was its inability to parse out the differential impact of majority vs. minority consensus feedback on conformist tendencies. Because we used responses from our prior sacred values study to provide the social feedback, rather than generating deceptive consensus data, the feedback was not normally distributed. The feedback was skewed such that the participants found themselves to be in agreement with the majority a disproportionate amount of the time. Thus, it is difficult to gauge whether our findings are generalizable to the condition in which participants find themselves to be in the minority much of the time.

The appraisal of one's attitudes and values appears to be a dynamic process. Given the potential for social pressure to influence this dynamic process, it is imperative that we better understand how social forces compel individuals to modify their attitudes, and what factors differentiate people in their susceptibility to social influence. Our research suggests that individual differences in conformist behavior surrounding one's personal attitudes and values are predicted by the deontological resolve behind one's sacred values.

## Supporting Information

Table S1 **Sacred and Non-Sacred Value Statements included in the Neuroimaging GLM of the Passive Phase.**
(DOCX)
