# Supplementary material for: The Conforming Brain and Deontological Resolve
Source: PLoS One. 2014 Aug 29;9(8):e106061. doi: 10.1371/journal.pone.0106061 (PMC4149480; doi:10.1371/journal.pone.0106061)
Supplement: Table S1 — Sacred and Non-Sacred Value Statements included in the Neuroimaging GLM of the Passive Phase. (DOCX) [file pone.0106061.s001.docx]

**Table S1.** Sacred and Non-Sacred Value Statements included in the Neuroimaging GLM of the Passive Phase.

**Sacred Values:**

| You would not give secret information about the US to a hostile foreign government. | You would give secret information about the US to a hostile foreign government. |
| --- | --- |
| You do not like to hurt animals. | You like to hurt animals. |
| You would not have sex with a woman. | You would have sex with a woman. |
| It is not ok to use nuclear weapons on civilians. | It is ok to use nuclear weapons on civilians. |
| You would not accept money for sex. | You would accept money for sex. |
| You would not cheat on your spouse even if there was no chance of getting caught. | You would cheat on your spouse if there was no chance of getting caught. |
| All whites are not racists. | All whites are racists. |
| You are not willing to kill an innocent human being. | You are willing to kill an innocent human being. |
| You believe in God. | You do not believe in God. |
| You think it is not ok to sell a child. | You think that it is ok to sell a child. |
| You think that homosexual couples should have the same rights as heterosexual couples. | You think that homosexual couples should not have the same rights as heterosexual couples. |
| You would not have sex with a 4 year old child. | You would have sex with a 4 year old child. |
| You would not have sex with a man. | You would have sex with a man. |

**Non-Sacred Values:**

| You believe that Google is superior to Yahoo. | You believe that Yahoo is superior to Google. |
| --- | --- |
| You are a dog person. | You are a cat person. |
| You are a tea drinker. | You are a coffee drinker. |
| You are a MAC person. | You are a PC person. |
| You have a favorite color M&M. | You enjoy all colors of M&M. |
| You do not drive above the speed limit. | You drive above the speed limit. |
| You are a Pepsi drinker. | You are a Coke drinker. |
| You believe that Wal-Mart is superior to Target. | You believe that Target is superior to Wal-Mart. |
| You prefer to watch Football over Basketball. | You prefer to watch Basketball over Football. |
| You would own a Hyundai before a Kia. | You would own a Kia before a Hyundai. |
| You are a white wine drinker. | You are a red wine drinker. |
